# Giant esophageal liposarcoma with squamous cell carcinoma resected via the cervical approach: a case report

**DOI:** 10.1186/s40792-022-01473-y

**Published:** 2022-06-20

**Authors:** Tomohiro Okura, Yasuhiro Shirakawa, Yuki Katsura, Takuya Yano, Michihiro Ishida, Daisuke Satoh, Yasuhiro Choda, Masanori Yoshimitsu, Nakano Kanyu, Hiroyoshi Matsukawa, Hitoshi Idani, Masazumi Okajima, Shigehiro Shiozaki

**Affiliations:** Department of Surgery, Hiroshima City Hiroshima Citizens Hospital, 7-33 Motomachi, Naka-ku, Hiroshima, 730-8518 Japan

**Keywords:** Esophagectomy, Verrucous carcinoma, Esophageal squamous cell carcinoma

## Abstract

**Background:**

Liposarcoma is one of the most common soft tissue sarcomas, but is extremely rarely found in the esophagus. There have been no reports of esophageal liposarcoma together with superficial carcinoma of the esophagus. Here, we report a patient who underwent complete resection of esophageal liposarcoma with carcinoma via a cervical approach.

**Case presentation:**

A 66-year-old man was diagnosed with an esophageal tumor 11 years ago, but he left it untreated. He presented to our hospital with progressive dysphagia and appetite loss since the previous year. Esophagogastroduodenoscopy (EGD) showed a large pedunculated submucosal tumor (SMT) originating at the esophageal entrance, extending to the gastroesophageal junction. Additionally, there was a superficial carcinoma on the surface of the SMT, 30 cm from the incisor teeth. Three-dimensional computed tomography (3D-CT) showed a giant elongated intraluminal tumor extending downwards from the cervical esophagus. We diagnosed a giant esophageal polyp accompanied by a superficial carcinoma and performed tumor resection via a cervical approach. The excised specimen consisted of a 23.0 × 8.5 cm polypoid mass. The final diagnosis by histopathological and immunohistochemical examination was well-differentiated liposarcoma and esophageal squamous cell carcinoma. He was discharged on postoperative day 14 with drastic improvement in his swallowing ability.

**Conclusion:**

We reported an extremely rare case of esophageal liposarcoma together with esophageal squamous cell carcinoma that was successfully resected through a small cervical incision.

## Background

Although liposarcoma is one of the most common soft tissue sarcomas in adults, representing approximately 20% of mesenchymal tumors [1], esophageal liposarcoma is extremely rare, accounting for 1.2 to 1.5% of all gastrointestinal liposarcomas [2]. Most esophageal liposarcomas present as an intraluminal mass with a long pedicle covered by intact mucosa. The condition is difficult to diagnose until the appearance of symptoms, including dysphagia, respiratory symptoms, and vomiting of the polypoid lesion through the mouth [3]. Symptomatic cases require surgical resection. To the best of our knowledge, 68 cases of esophageal liposarcoma have been reported since 1983, when the first case was reported [4–6]. However, there have been no reports of esophageal liposarcoma accompanied by esophageal carcinoma. Here, we report the first case of a patient who underwent complete resection of an esophageal liposarcoma along with a superficial carcinoma via a cervical approach.

## Case presentation

**Fig. 1:**
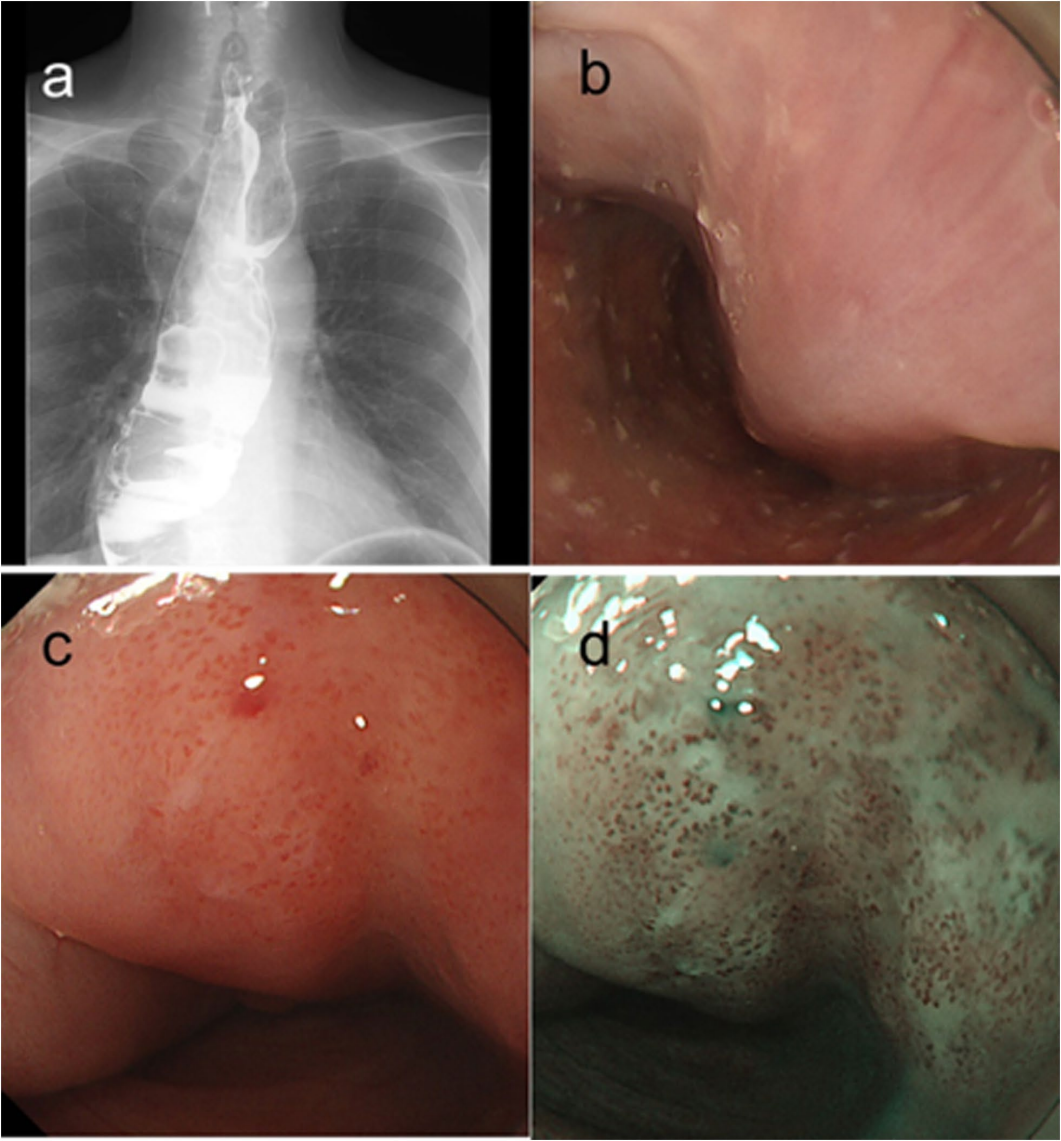
Preoperative upper gastrointestinal imaging and esophagogastroduodenoscopy. **a**, **b** A giant tumor was seen, arising from the posterior wall of esophageal entrance and extending to the gastroesophageal junction, with the majority of the surface covered with normal mucosa. **c**, **d** There was a localized erythematous lesion on the tumor, and type B1 vessels were identified

**Fig. 2:**
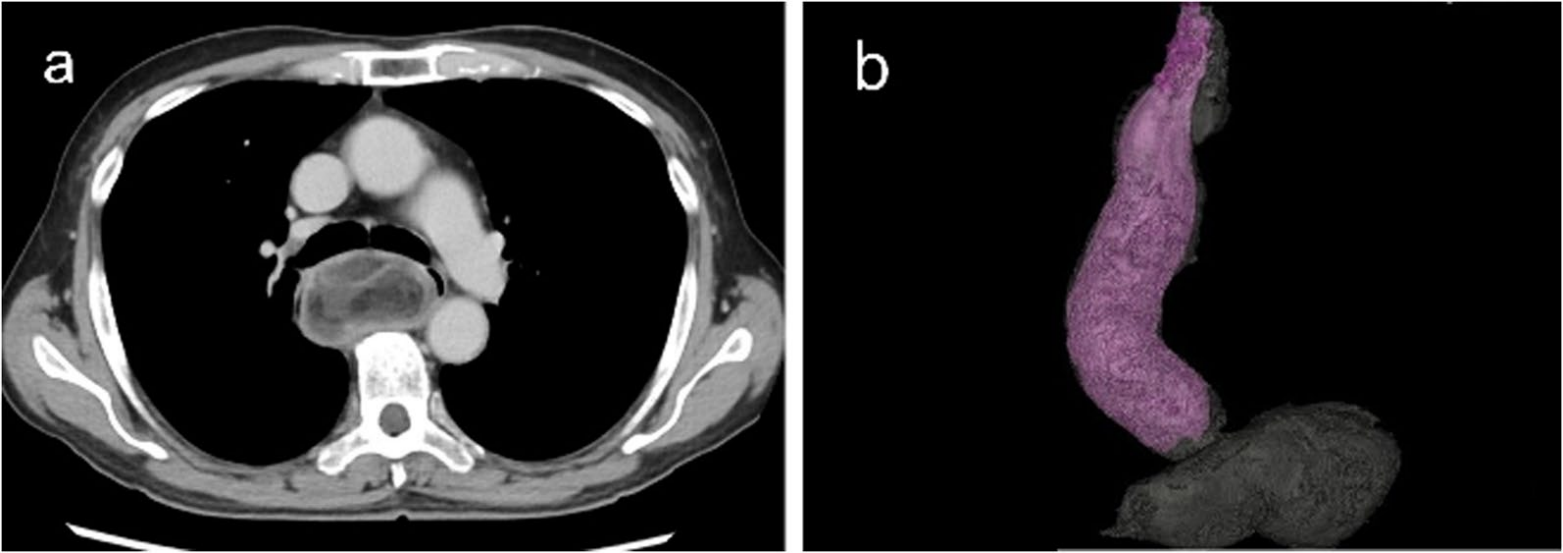
**a** Preoperative contrast-enhanced CT showed a large pedunculated tumor hanging in the cervical esophagus. The inside of the tumor was heterogeneously contrasted, with a mixture of fatty, fibrous and vascular components. **b** 3D-CT showed that the tumor was an intraluminal polypoid tumor

**Fig. 3:**
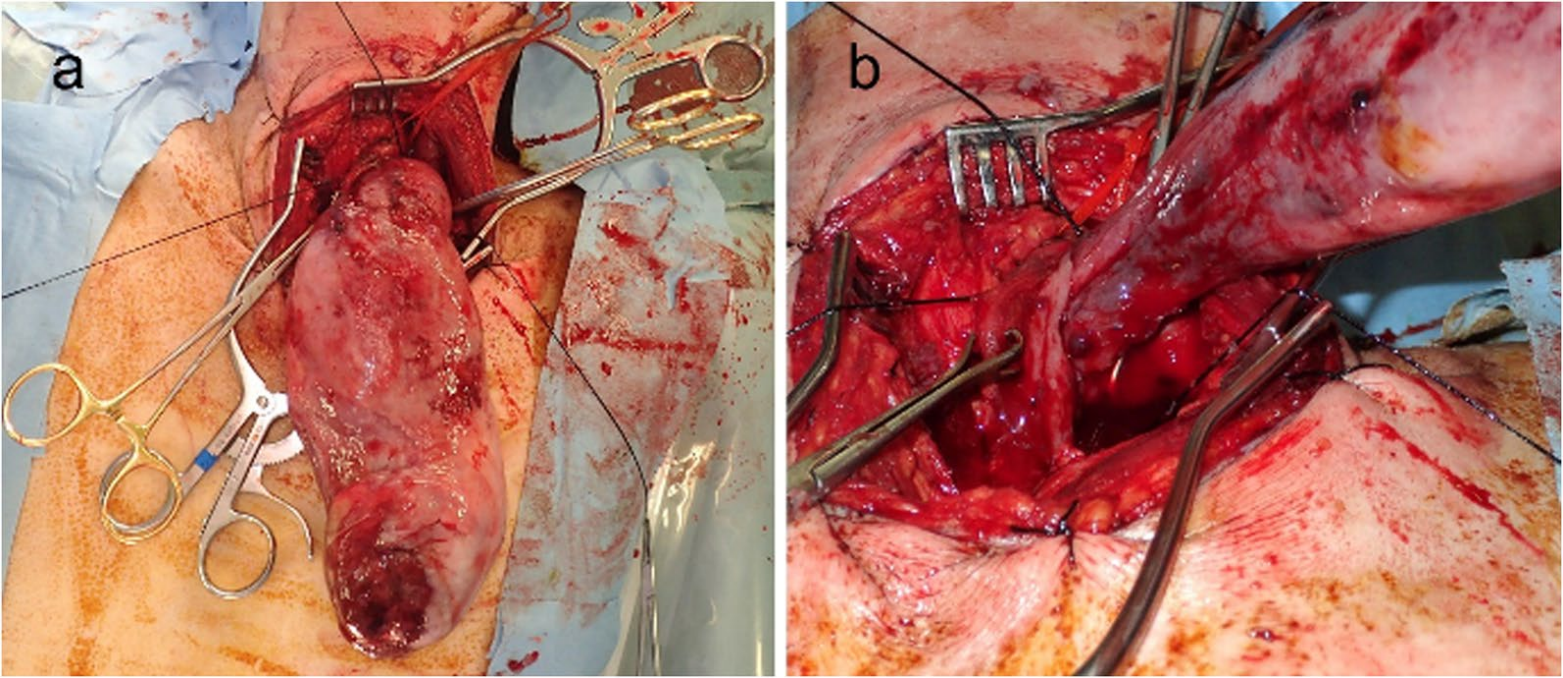
Intraoperative view. **a** A 7-cm skin incision was made on the left side of the neck. The giant tumor was directly grasped and carefully pulled out of the esophageal lumen. **b** The stalk of the tumor arising from the posterior wall was transected and the tumor was extracted

A 66-year-old man underwent high anterior resection for rectal cancer 11 years ago. He had also been diagnosed with an esophageal tumor 15 cm in size at the same time, based on the symptom of mild dysphagia, but had left the tumor untreated. Recently, he presented to our hospital with progressive dysphagia and appetite loss, which had gradually worsened over the course of the previous year. He had no history of smoking or drinking alcohol. His tumor markers were not increased (carcinoembryonic antigen: 2.0 ng/mL, squamous cell cancer antigen: 0.9 ng/mL). Upper gastrointestinal imaging (UGI) and esophagogastroduodenoscopy (EGD) showed a large tumor with a thick stalk arising from the esophageal entrance, extending to the gastroesophageal junction, with a normal surface mucosa (Fig. 1a, b). Since we could not identify details of this submucosal tumor (SMT), we considered it a so-called fibrovascular polyp. Furthermore, a localized erythematous lesion and type B1 vessels according to the Japan Esophageal Society classification [7] were identified on the tumor surface, 30 cm from the incisor teeth (Fig. 1c, d). Contrast-enhanced computed tomography (CT) showed a large tumor hanging from the cervical esophagus, extending to the gastroesophageal junction (Fig. 2a). The inside of the tumor was heterogeneously contrasted with a mixture of fatty, fibrous and vascular components. The length of the tumor was about 30 cm, indicating that it had doubled in size over the last 10 years. There were no findings suggesting lymph node or distant metastasis. Three-dimensional CT (3D-CT) revealed that the tumor was an intraluminal polypoid tumor (Fig. 2b). Our preoperative diagnosis was a so-called giant fibrovascular polyp with superficial carcinoma of the esophagus. From the above findings, we determined that it was possible to achieve radical en bloc resection of the tumor through the cervical approach as a minimally invasive procedure, without lymph node dissection. With the patient under general anesthesia, we made a 7-cm skin incision on the left side of the neck. We cut the lateral side of the anterior cervical muscles, but preserved the sternocleidomastoid muscle and entered the inner side of the common carotid artery. After that, we reached the wall of the esophagus and taped it. After directly incising the esophageal wall at the opposite side of tumor peduncle and encircling the stalk of the tumor, the giant tumor was directly grasped and could be carefully pulled out of the esophageal lumen (Fig. 3a). We transected the stalk of the tumor arising from the posterior wall and extracted it (Fig. 3b). The basal mucosal layer of the esophagus was closed using continuous 4–0 absorbable sutures, and the muscle layer was closed with nodal sutures using 3–0 absorbable sutures. The surgical duration was 2 h and 22 min, and blood loss was minimal. The patient had an uneventful postoperative course without any complications, and was discharged on postoperative day 14 without dysphagia.

**Fig. 4:**
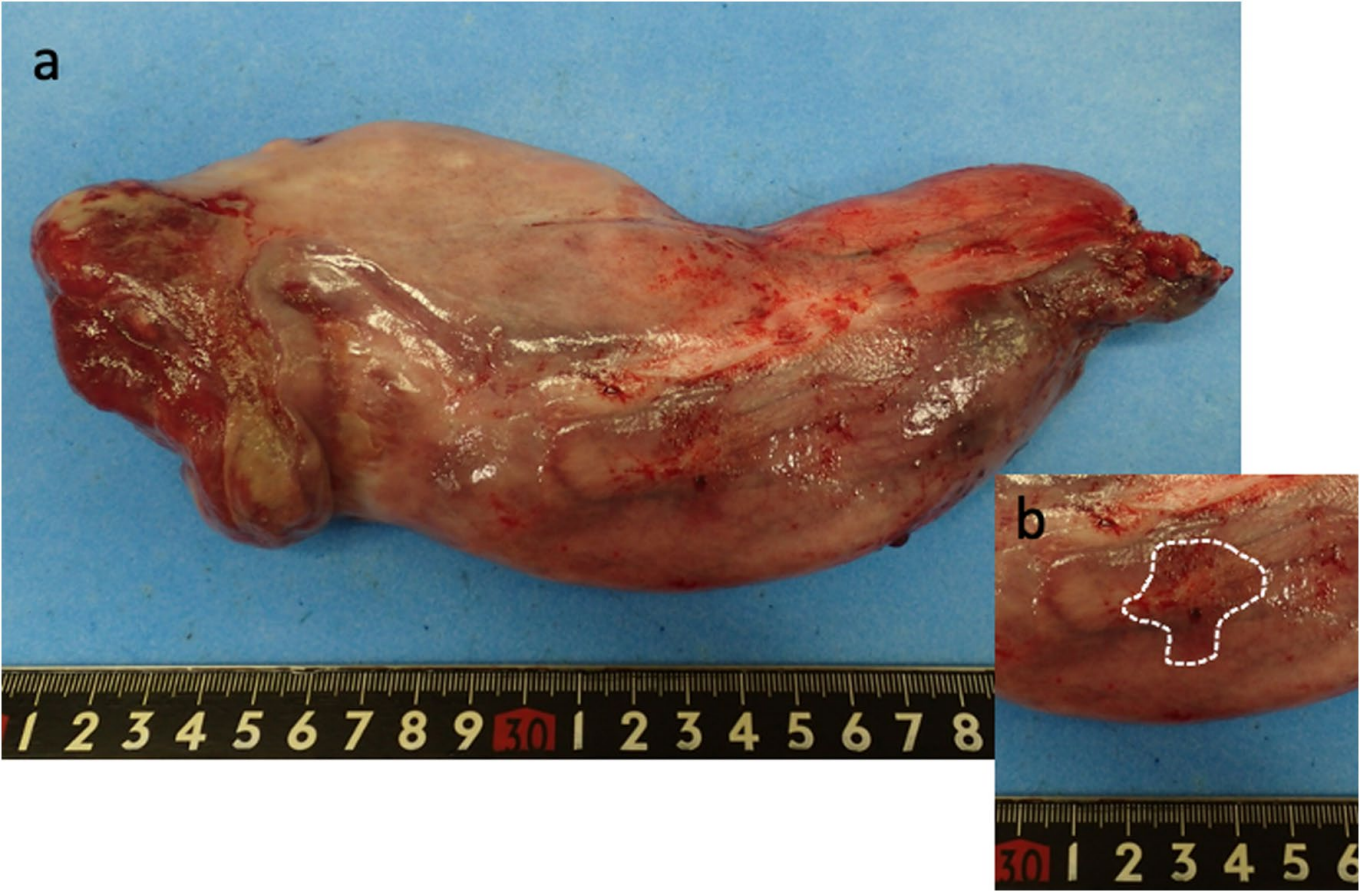
The excised specimen. **a** Overview of a 23.0 × 8.5 cm polypoid mass. **b** Superficial carcinoma located on the surface of the tumor

**Fig. 5:**
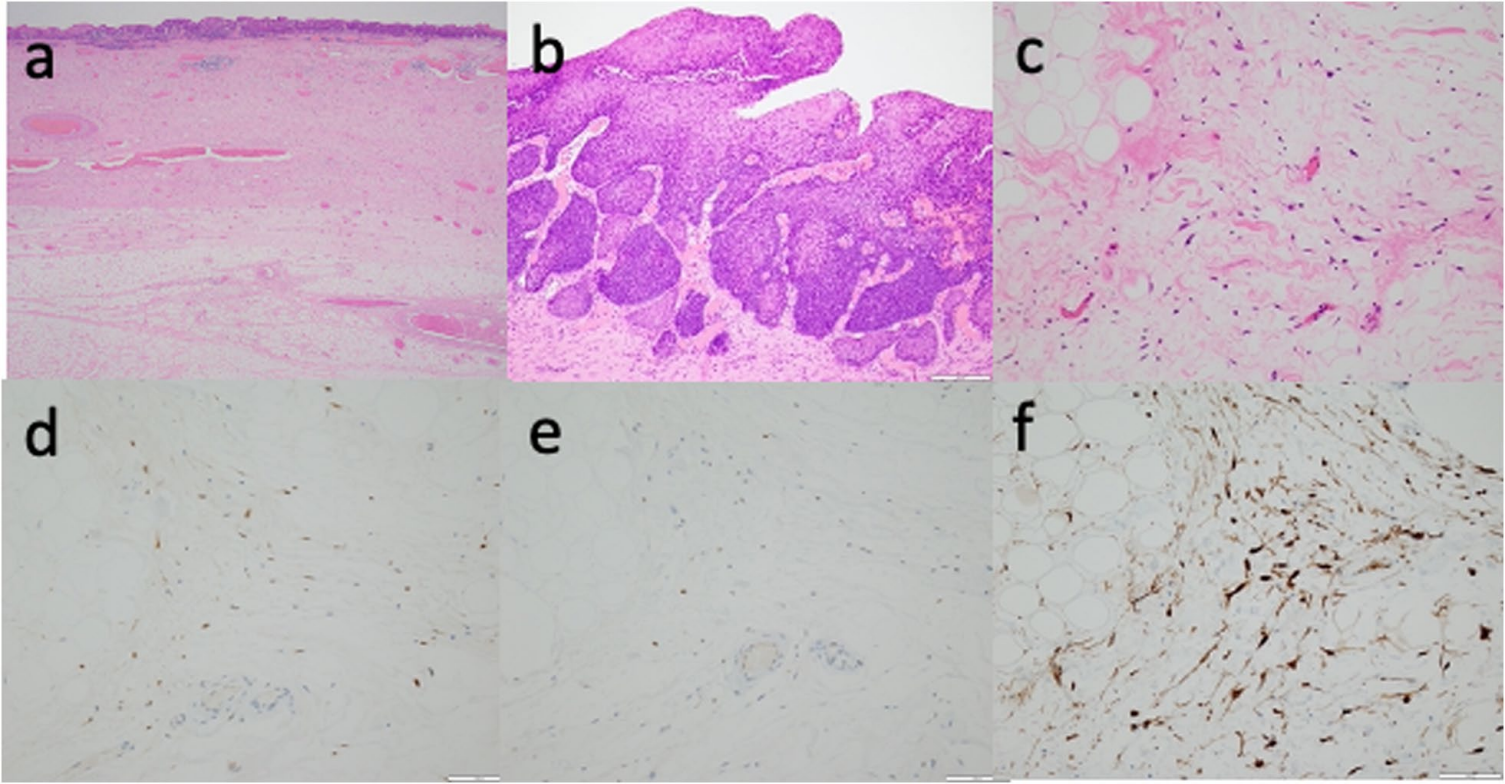
Histopathological examination. **a** superficial squamous carcinoma located on the surface of liposarcoma. **b** Proliferation of atypical squamous epithelium cells and invasion into the stroma in a part of the tumor. The depth of invasion was 930 μm. **c** Most tumors were composed of adipocytes and spindle cells with atypical nuclei. (**a** 1 × , **b** 10 × , and **c** 20 × , hematoxylin and eosin staining). **d**, **e**, **f** Immunostaining showed atypical lipoblasts positive for CDK4 (**d**), weakly positive for MDM2 (**e**), and positive for p16 in the nuclei (**f**). (**d**, **e**, and **f** All 20 ×)

The excised specimen consisted of a 23.0 × 8.5 cm polypoid mass and superficial esophageal carcinoma located on it (Fig. 4a, b). Histological examination showed proliferation of squamous cell carcinoma in the epithelium and invasion of the stroma in some areas, although there was no lamina muscularis mucosa. The depth of invasion was 930 μm from the surface of mucosa (Fig. 5a, b), and there was no vascular or lymphatic invasion. On the other hand, most of the submucosal tumor was composed of adipocytes and spindle cells with atypical nuclei (Fig. 5a, c). Immunohistochemical analysis showed that the adipocytes were weakly positive for murine double minute-2 (MDM2) and positive for cyclin-dependent kinase 4 (CDK4) and p16 (Fig. 5d, e, f). The final pathological diagnosis was well-differentiated esophageal liposarcoma together with squamous cell carcinoma. The surgical margin of the tumor was microscopically negative. The patient did not undergo any postoperative adjuvant therapy and gained 10 kg in weight within 3 months postoperatively.

## Discussion

Liposarcoma is one of the most common soft tissue sarcomas in adults, representing around 20% of mesenchymal tumors. It usually occurs in the retroperitoneum, deep soft tissues of the trunk, or lower extremities [1, 8]. Primary gastrointestinal liposarcomas are much rarer, with a reported incidence at autopsy between 0.1 and 5.8% [9, 10]. Esophageal liposarcoma is extremely rare, accounting for 1.2 to 1.5% of all gastrointestinal liposarcomas [2]. Esophageal liposarcoma was reported for the first time in 1983 [4]. As far as we know, there have been 68 reported cases of this tumor so far. However, there have been no reports of esophageal liposarcoma together with esophageal carcinoma [5, 6]. Liposarcoma originates from primitive mesenchymal cells, and usually arises from the esophageal submucosa [11, 12]. On the other hand, since esophageal carcinoma arises from the esophageal epithelium, their origins are different. There have been some reports of gastrointestinal stromal tumor (GIST) combining gastric cancer regarding mesenchymal tumors other than liposarcoma [13, 14]. According to those reports, there was no correlation between the gastric carcinogenesis and GIST. In our case, most of the tumor was covered by normal mucosa, and esophageal carcinoma was only found in a localized area. This suggests that although the two tumors were contiguous with each other, they should be considered as separate entities with distinct occurrences. This case is the first report of the coexistence of esophageal liposarcoma and esophageal carcinoma.

Esophageal liposarcomas can lead to esophageal obstruction following their slow growth to a large size. Patients usually present with symptoms such as dysphagia, cough, vomiting, foreign body sensation in the throat, airway obstruction, and even asphyxiation [10, 15]. Tumor resection is the only curative treatment for esophageal liposarcoma [16, 17]. Classically, open surgery has been the standard treatment, although in recent years, endoscopic resection has become a promising option for tumor resection. If the tumor is relatively small, pedunculated, has a single stalk, and with no large vessels in the stalk, endoscopic resection is considered to be a good treatment option [15]. However, when the tumor is too large to be removed orally after endoscopic resection, it requires esophagotomy through a cervical incision [18]. In cases where endoscopic resection is not an option, open surgery facilitates definitive resection, including partial or total esophagectomy. However, these surgeries are more invasive procedures, requiring an extended postoperative hospital stay. Hence, in cases in which the tumor develops from the cervical esophagus, a cervical approach can be an effective option [15].

There have been six previous reports of cases of esophageal liposarcoma that were completely resected and removed only through a cervical incisional approach [4, 6, 19–22] (Table 1). Among them, five cases underwent surgery via a left cervical approach, with the largest tumor being 24 cm in diameter. However, there are some limitations to this procedure. First, it is essential to evaluate the precise location of the tumor stalk in the cervical esophagus by preoperative imaging. Second, if the tumor is too large, it might compress the trachea when it is pulled out of the esophagus, leading to airway obstruction. In such cases, the tumor might need to be pulled out from the abdominal esophagus via a laparotomy. Third, the cervical approach is not suitable for cases where mediastinal lymph node dissection is needed, because it is difficult to perform such dissection via the cervical approach. In the present case, we confirmed that the tumor stalk was located in the cervical esophagus by preoperative 3D-CT. Additionally, although esophageal carcinoma was suspected, lymph node dissection was not deemed necessary because it was considered to be a superficial cancer. Referencing the tumor sizes in the previous cases, we judged that they were all completely resectable by a cervical approach.

**Table 1:** Literature review of seven cases, including our case, of esophageal liposarcoma that were removed only through a cervical incision

	Year of publication	Age (y)/sex	Type of lesion	Presenting symptom	Approach	Tumor size (cm)	Histology
Mansour [4]	1983	53/M	Polypoid	Dysphagia	Right cervical	4 × 3	Myxoid
Salis [20]	1998	73/M	Polypoid	Dysphagia, vomiting	Left cervical	15 × 6	Well-differentiated
Maruyama [24]	2007	50/M	Polypoid	Cough	Left cervical	18.5 × 8.5 × 4	Well-differentiated
Saleh. [19]	2013	62/M	Polypoid	Dysphagia, weight loss	Left cervical	24	Well-differentiated
Dowli [15]	2014	64/M	Polypoid	Dysphagia	Left cervical	15 × 7 × 3	Well-differentiated
Furukawa [6]	2021	72/F	Polypoid	Dysphagia	Left cervical	15 × 7 × 5	Well-differentiated
Our case	2021	66/M	Polypoid	Dysphagia	Left cervical	23 × 8.5	Well-differentiated

Tumor aggressiveness and the prognosis of esophageal liposarcoma are still unclear, but might depend on the histological type, location, and status of surgical margins [6, 23]. Ferrari D, et al. reported six cases of esophageal liposarcoma recurrences. The recurrence was locoregional in all cases, and two of them resulted in tumor-related death [5]. However, the well-differentiated type of esophageal liposarcoma, previously categorized as a fibrovascular polyp, has recently been reported to have the highest 5-year survival rate and the lowest local recurrence rate [24]. Therefore, tumors of this histological type should be treated with complete surgical resection, which is likely to result in an excellent prognosis. In our case, the histological type was well-differentiated, and the surgical margin was microscopically negative. Thus, the risk of liposarcoma recurrence is likely low. However, long-term follow-up with EGD is necessary in our patient because of the possibility of a new esophageal carcinoma in the remnant esophagus.

## Conclusions

We report an extremely rare case of esophageal liposarcoma accompanied by superficial esophageal squamous cell carcinoma. We successfully resected the giant tumor through a left cervical approach. A giant liposarcoma needs to be treated with surgical resection. In such cases, preoperative 3D-CT is helpful for determining the surgical site and process. The cervical approach is useful when it is clear that the tumor stalk is in the cervical esophagus.

## Data Availability

Data sharing is not applicable to this article as no datasets were generated or analyzed during the current study.

## Abbreviations

UGI: Upper gastrointestinal imaging
ESD: Esophagogastroduodenoscopy
SMT: Submucosal tumor
3D-CT: Three-dimensional computed tomography
CT: Computed tomography
MDM2: Murine double minute-2
CDK4: Cyclin-dependent kinase 4
GIST: Gastrointestinal stromal tumor
